# Remote ischemic preconditioning improves tissue oxygenation in a porcine model of controlled hemorrhage without fluid resuscitation

**DOI:** 10.1038/s41598-021-90470-6

**Published:** 2021-05-24

**Authors:** Gal Yaniv, Arik Eisenkraft, Lilach Gavish, Linn Wagnert-Avraham, Dean Nachman, Jacob Megreli, Gil Shimon, Daniel Rimbrot, Ben Simon, Asaf Berman, Matan Cohen, David Kushnir, Ruth Shaylor, Baruch Batzofin, Shimon Firman, Amir Shlaifer, Michael Hartal, Yuval Heled, Elon Glassberg, Yitshak Kreiss, S. David Gertz

**Affiliations:** 1 Institute for Research in Military Medicine (IRMM), Faculty of Medicine, The Hebrew University of Jerusalem and the Israel Defense Forces Medical Corps, POB 12272, 91120 Jerusalem, Israel; 2 Sheba Medical Center, Tel-Hashomer, Ramat-Gan, Israel; 3 The Saul and Joyce Brandman Hub for Cardiovascular Research and the Department of Medical Neurobiology, Institute for Medical Research (IMRIC), Faculty of Medicine, The Hebrew University of Jerusalem, Jerusalem, Israel; 4 Heart Institute, Hadassah Hebrew University Medical Center, Jerusalem, Israel; 5 Israel Defense Forces Medical Corps, Jerusalem, Israel; 6 Center for Innovative Surgery, Hadassah Medical Center, Jerusalem, Israel; 7 Division of Anesthesiology and Intensive Care, Tel-Aviv Sourasky Medical Center, Tel-Aviv, Israel; 8 The Department of Anesthesiology, Hadassah Medical Center, Jerusalem, Israel; 9 The Myers-JDC-Brookdale Institute, Jerusalem, Israel; 10 The Uniformed Services University of the Health Sciences, Bethesda, MD USA; 11 The Azrieli Faculty of Medicine, Bar-Ilan University, Safed, Israel

**Keywords:** Medical research, Signs and symptoms

## Abstract

Remote ischemic preconditioning (RIPC) involves deliberate, brief interruptions of blood flow to increase the tolerance of distant critical organs to ischemia. This study tests the effects of limb RIPC in a porcine model of controlled hemorrhage without replacement therapy simulating an extreme field situation of delayed evacuation to definitive care. Twenty-eight pigs (47 ± 6 kg) were assigned to: (1) control, no procedure (n = 7); (2) HS = hemorrhagic shock (n = 13); and (3) RIPC + HS = remote ischemic preconditioning followed by hemorrhage (n = 8). The animals were observed for 7 h after bleeding without fluid replacement. Survival rate between animals of the RIPC + HS group and those of the HS group were similar (HS, 6 of 13[46%]-vs-RIPC + HS, 4 of 8[50%], *p* = 0.86 by Chi-square). Animals of the RIPC + HS group had faster recovery of mean arterial pressure and developed higher heart rates without complications. They also had less decrease in pH and bicarbonate, and the increase in lactate began later. Global oxygen delivery was higher, and tissue oxygen extraction ratio lower, in RIPC + HS animals. These improvements after RIPC in hemodynamic and metabolic status provide essential substrates for improved cellular response after hemorrhage and reduction of the likelihood of potentially catastrophic consequences of the accompanying ischemia.

## Introduction

Hemorrhage remains to be the principal cause of preventable death on the battlefield^1,2^. A variety of technologies have been directed toward control of bleeding including tourniquets, direct pressure, hemostatic dressings, and circulatory volume replacement^3,4^. Although these techniques have improved with time, the fundamental principles behind these standard approaches have not changed substantially since World War-II^5,6^. Remote Ischemic preconditioning (RIPC) involves deliberate, brief, repetitive periods of interruption of blood flow followed by reperfusion. It is designed to be performed in an organ that is not immediately damaged by reduction of blood supply such as a limb. The rationale is to increase the tolerance to ischemia of distant critical organs such as the heart, brain, or kidney^7–9^. A variety of studies have been concerned with the identification and characterization of circulating factors that mediate these remote effects^10,11^. We hypothesized that RIPC could be used as a simple, non-invasive, pre-mission approach to improve survivability in the event of traumatic hemorrhage during combat.

Although protective effects of RIPC have been reported in numerous animal models, its application to the human interventional setting produced conflicting results–some of which were attributed to the anesthesia regimen used in these studies^12–15^. The potential of RIPC to protect against damage induced by hemorrhagic shock was studied in rodents by several groups. All showed positive effects including decreased levels of pro-inflammatory cytokines^16,17^, mitigation of lung injury^16,18^ and lung edema^17^, improved survival^19,20^, improved myocardial and neurologic function^19^, improved blood pressure^20^, and decreased lactate production^17^. See recent review by Kloner et al.^21^. However, in all of these animal studies, the induced shock was followed by fluid resuscitation.

**Figure 1:**
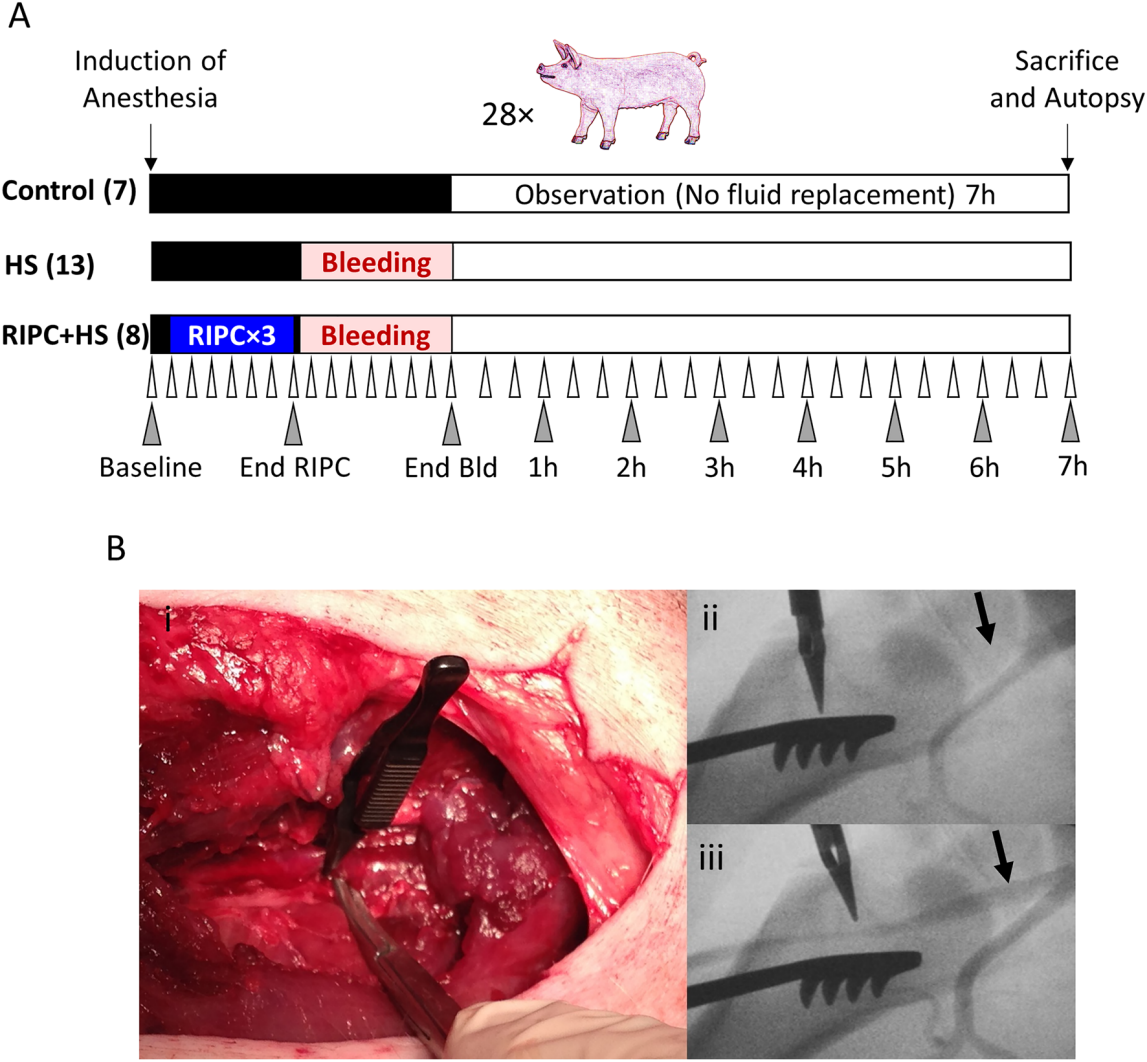
Study Design and Remote Ischemic Preconditioning Procedure. (**A**) Study Design RIPC = remote ischemic preconditioning; HS = hemorrhagic shock; Bld = Bleeding; White triangles: hemodynamic measurements every 5 min from baseline to end of HS and every 20 min during the observation period; Grey triangles: blood/urine sample collection. (**B**) i. Occlusion of the right femoral artery by bulldog clamp; ii. Angiography demonstrating occlusion of femoral artery blood flow, and iii. Reperfusion of the femoral artery after removal of the clamp. Black arrows point to the femoral artery branching from the iliac.

In a previous study of controlled hemorrhagic shock in a large animal (porcine) model, we reported that maladaptive responses across a range of cardiovascular parameters that begin early after hemorrhage may be predictive of impending death, particularly in situations where early resuscitative treatment may be delayed^22^. The current study was designed to test the effect of limb RIPC on survival, as well as a broad range of hemodynamic and biochemical parameters, in this large animal model of controlled hemorrhage, without fluid return or replacement therapy (Fig. 1), simulating that which may occur in extreme field situations involving several hours of delayed evacuation and definitive medical care.

## Results

### Accountability

The % blood volume withdrawn to reach hemorrhagic shock was the same for animals that underwent RIPC (RIPC + HS) as those that did not (% blood volume withdrawn, median [IQR]: HS, 35.0[2.0]% -vs- RIPC + HS, 34.5[4.5]%, *p* = 0.55 by Exact MWU-test).

### Survival

The percentages of animals that survived to the end of the 7-h follow-up period between those of the RIPC + HS group and those of the HS only group were similar (#died of total pigs: RIPC + hemorrhagic shock (HS), 4 of 8 [50%] -vs- HS, 6 of 13 [46%], *p* = 0.86 by chi-square). The Kaplan–Meier Survival plot showed no significant difference in mean survival time (MST) between groups (MST [95% Confidence Intervals]: RIPC + HS, 362 min [322–401] -vs- HS, 337 min [275–399]) (Supplemental Figure S1). Nevertheless, overall, the animals of the RIPC + HS group had better results than those without RIPC in several important parameters as detailed below. This was the case whether considering all animals together or restricting the analysis to just those that survived through the follow-up.

### Acute effects of RIPC

RIPC alone, prior to bleeding, had no apparent effect on any of the tested baseline clinical or laboratory parameters (Supplemental Table S1).

### Hemodynamics (Table 1)

**Table 1:** Hemodynamic parameters.

Variable	Control (C)			HS (H)			RIPC + HS (R)			*p* Value between groups		
	Base	EndBld	FU	Base	EndBld	FU	Base	EndBld	FU	Comparison	ΔBld	ΔFU
MAP [mmHg]										C/H	< 0.001	0.002
	64 ± 10	61 ± 10	56 ± 12	67 ± 11	29 ± 6†	36 ± 11†	67 ± 8	30 ± 3†	42 ± 8†‡	C/R	< 0.001	< 0.001
				67 ± 12	30 ± 7†	41 ± 10†‡	69 ± 5	29 ± 1†	46 ± 2†‡	H/R	NS	NS
HR [bpm]										C/H	0.02	0.005*
	89 ± 12	82 ± 12	93 ± 18‡	83 ± 9	105 ± 27	126 ± 30†‡	82 ± 17	127 ± 40†	166 ± 25†‡	C/R	0.001	< 0.001*
				85 ± 7	103 ± 24	124 ± 17†‡	81 ± 17	128 ± 40	163 ± 28†	H/R	0.007	0.003*
CI [l/min/m^2^]										C/H	< 0.001	0.055
	3.2 ± 0.9	3.3 ± 0.8	3.1 ± 1.0	3.9 ± 0.4	1.8 ± 0.5†	2.1 ± 0.6†	3.7 ± 0.7	1.9 ± 0.5†	2.4 ± 0.7†	C/R	< 0.001	0.017
				3.9 ± 0.4	1.9 ± 0.6†	2.4 ± 0.5†	3.7 ± 0.7	1.9 ± 0.6†	2.6 ± 0.7‡	H/R	NS	NS
SV [ml]										C/H	< 0.001	0.007
	35 ± 11	39 ± 10	33 ± 10	43 ± 5	16 ± 5†	16 ± 4†	42 ± 11	15 ± 6†	14 ± 5†	C/R	< 0.001	0.040
				42 ± 5	17 ± 4†	18 ± 4†	43 ± 13	14 ± 7†	15 ± 6†	H/R	NS	NS
PCWP [mmHg]										C/H	–^a^	–^a^
	9.9 ± 6	8.7 ± 5.3	8.9 ± 4.9	7.8 ± 4.2	4.6 ± 4.0†	5.0 ± 3.5†	9.1 ± 5.4	6.5 ± 3.1	5.7 ± 2.8	C/R	–	–
				7.7 ± 4.7	4.0 ± 3.6†	5.0 ± 4.0†	9.4 ± 5.9	7.4 ± 2.4	5.5 ± 2.5	H/R	–	–

Data presented as mean ± SD. Numbers in first row of each parameter are for all animals. Numbers in second row of each parameter are only those that survived (6 h of follow-up: Controls, n = 7 at all time-points; HS, n = 13 at 2H, n = 10 at 4H, n = 9 at 6H; RIPC + HS, n = 8 at 2H and 4H, n = 5 at 6H). Normality was determined by Shapiro–Wilk (SW) test (*p* > 0.1). Comparisons between baseline, end of bleeding [EndBld], and follow-up [FU] (mean over 7-h follow-up) within groups were performed by paired t-test with Bonferroni’s correction for multiple comparisons or Friedman’s test with pairwise comparisons as post-hoc test as appropriate (according to SW). Comparisons between groups (relates to all 7 h) were performed by analysis of variance (ANOVA) with Fisher’s least significance difference (FLSD) as post-hoc test or Kruskal–Wallis (KW) with Conover-Iman as *post-hoc* test. MAP = mean arterial pressure, HR = heart rate, CI = cardiac index, SV = stroke volume, PCWP = Pulmonary capillary wedge pressure. ΔBld = (EndBld-Baseline), ΔFU = (FU-EndBld), *ΔFU = (FU-Baseline). *p* < 0.05 considered significant and all tests 2-tailed. †*p* < 0.05 versus baseline; ‡*p* < 0.05 versus EndBld; ^§^not significant by ANOVA/KW.

#### Mean arterial pressure

**Figure 2:**
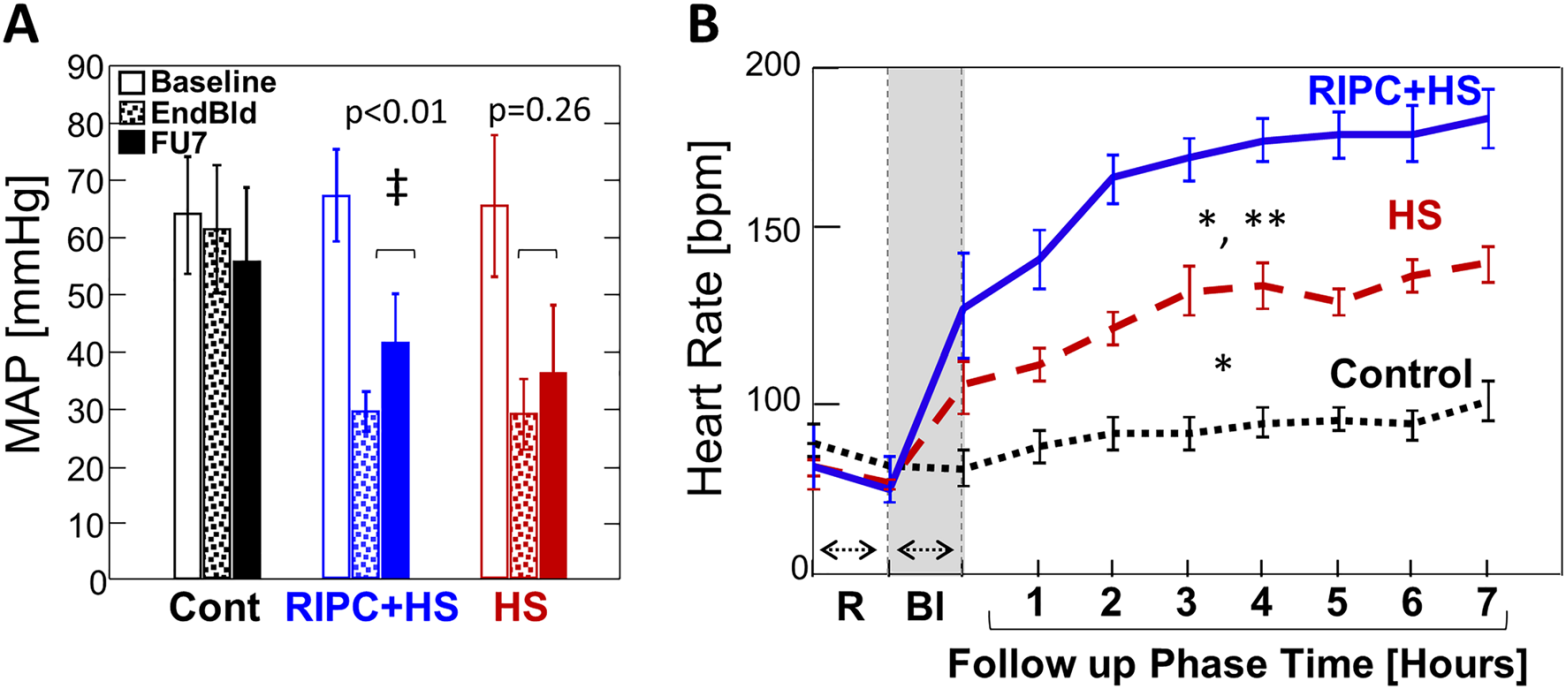
Hemodynamics: MAP and Heart Rate. (**A**) Mean Arterial Pressure (MAP) Compensation: Bars represent mean ± SD. Note significant increase in MAP in the follow-up period compared to the end of bleeding in animals with prior RIPC (RIPC + HS) but not in animals without RIPC (HS only). ‡*p* < 0.05 by paired t-test with Bonferroni’s correction for multiple comparisons (**B**) Heart Rate (HR): Data points and error bars represent mean ± SEM. Note: animals of RIPC + HS group had greater compensation of HR after bleeding and during the follow-up period. R = time of RIPC, Bl = time of bleeding [grey rectangle]. *p* < 0.05 * versus Control; ** versus HS by ANOVA with FLSD as *post-hoc* test. #animals: Controls n = 7; HS n = 13; RIPC + HS n = 8.

**Figure 3:**
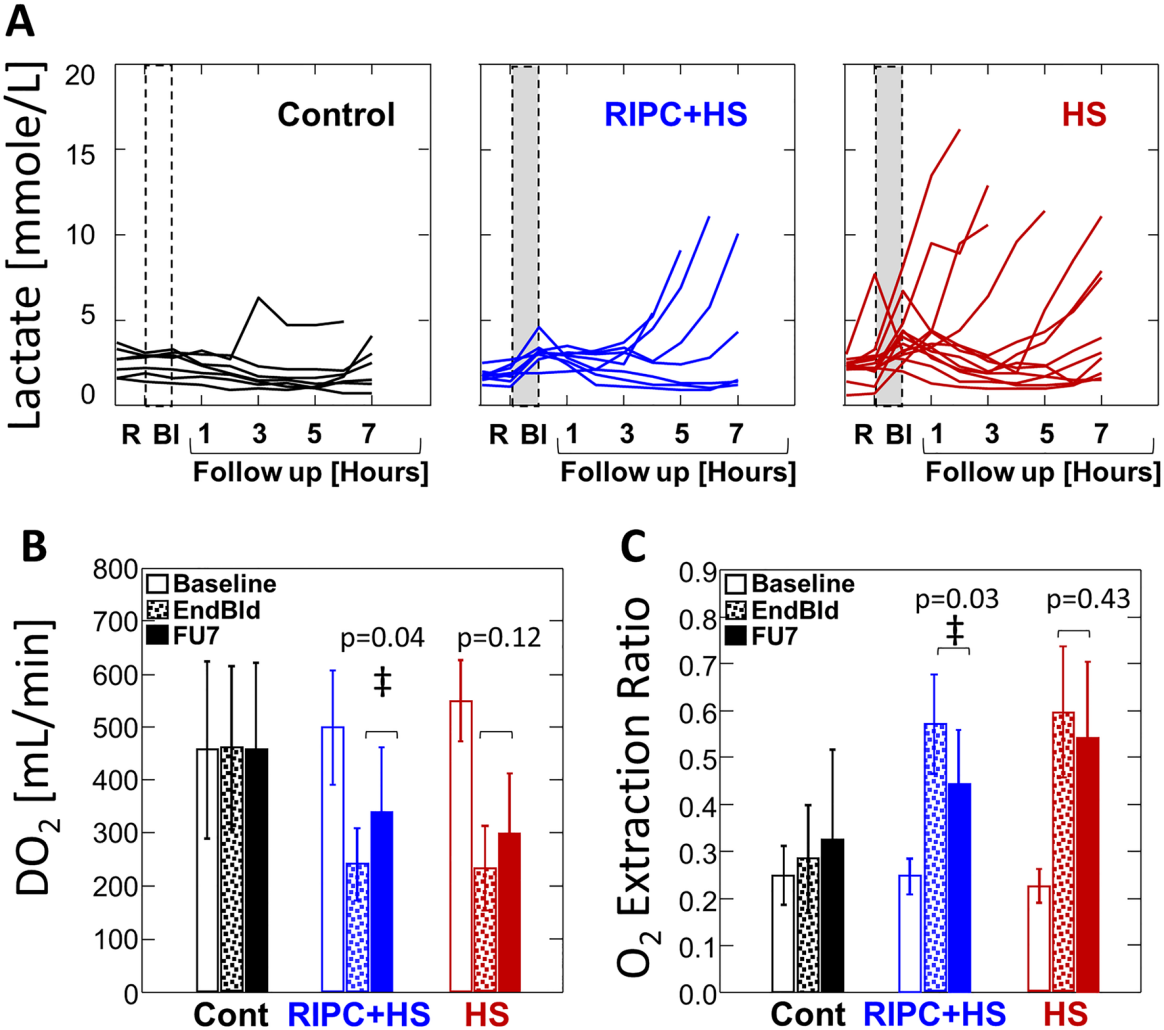
Analysis by Survivors. Change in mean arterial pressure (MAP), cardiac index (Cardiac Ind.), global oxygen delivery (DO_2_), and oxygen extraction ratio for controls, animals that underwent bleeding (HS) and those that received RIPC prior to bleeding (RIPC + HS) at 2, 4, and 6 h follow-up compared to end of bleeding restricted to animals that survived at each time. #animals: Controls: n = 7; HS n = 13, 10, 9 at 2 h, 4 h, 6 h; RIPC + HS n = 8, 8, 5 at 2 h, 4 h, 6 h.

The study protocol required a set mean arterial pressure (MAP) of 30 mmHg as the endpoint for bleeding, and therefore, as expected, there was no significant observed difference in MAP at this time point between the two groups that were bled (Table 1) (Note: the mean MAP for normal control young, anesthetized pigs in this study was similar to that reported by others [64 ± 10 mmHg, see Table 1]). However, animals of the RIPC + HS group had a significant increase in MAP during follow-up period (FU) compared to the end of bleeding (*p* = 0.01). This was not observed in animals of the HS only group (*p* = 0.26, Fig. 2A). When restricting the analysis to those that survived at 6 h follow-up (Table 1, Fig. 3, 5 of 5 (100%) animals in the RIPC + HS group had at least 50% increase in MAP, while only 3 of 9 (33%) animals of the HS group reached the same level of compensation during follow-up (*p* = 0.031 by FET).

#### Heart rate

Animals of the RIPC + HS group developed a significant increase in heart rate by the end of bleeding (*p* = 0.007) that reached a 50% or more increase over baseline during the 7-h follow-up (Table 1, Fig. 2B). However, in the HS only group, less than half the animals reached this degree of increase in heart rate (*p* = 0.015). Likewise, restricting the analysis to those that survived follow-up also showed that RIPC + HS animals maintained a significantly increased HR at the end of follow-up compared to HS only animals (*p* = 0.001) (Table 1).

#### Stroke volume, pulmonary capillary wedge pressure, and cardiac index

At the end of bleeding, all animals had initial prominent reduction in stroke volume (SV) and pulmonary capillary wedge pressure (PCWP) without additional changes during follow-up period, and without significant changes between animals with or without RIPC. The difference in cardiac index (CI) between animals bled with or without RIPC between end of bleeding and follow-up did not reach significant statistical separation (Table 1). When restricting the analysis to those that survived, the cardiac index of the RIPC + HS group was significantly greater at the end of follow-up versus end of bleeding (*p* = 0.029), but this recovery was not observed in the HS-only group (Table 1, Fig. 3).

### Tissue oxygenation and acid base homeostasis

**Figure 4:**
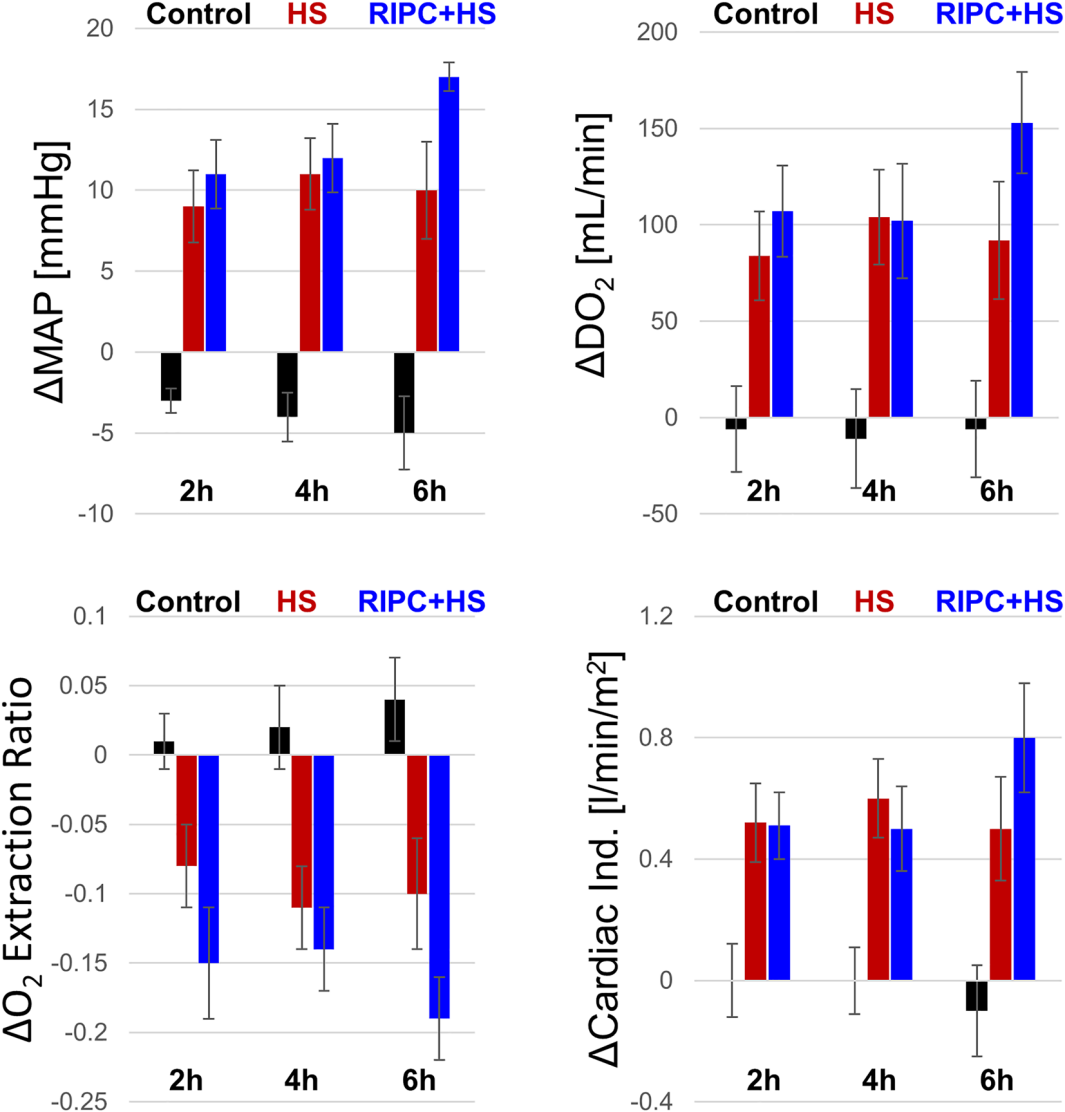
Lactate and Tissue Oxygenation. (**A**) Lactate: Measurements of individual animals for each group. Note the delayed rise in lactate in animals with RIPC prior to bleeding (RIPC + HS) versus those bled without RIPC (HS). (**B**, **C**) Tissue Oxygenation: Bars represent mean ± SD. Note significant increase in global oxygen delivery (DO_2_) and decrease in tissue oxygen extraction ratio (ER) in follow-up period compared to the end of bleeding in animals of RIPC + HS group but not in animals of HS group. ‡*p* < 0.05 by paired t-test with Bonferroni’s correction for multiple comparisons. #animals: Controls n = 7; HS n = 13; RIPC + HS n = 8.

The ventilation protocol administered during the experiment was designed to achieve constant End-tidal CO_2_. Thus, any change in acid–base homeostasis would likely be of metabolic origin. The decrease of both pH and bicarbonate was more prominent in the animals of the HS only group, with a significantly smaller change in base excess (BE) in the animals of the RIPC + HS group (Table 2). Lactate levels in animals of the HS only group were significantly higher after the end of bleeding and during the follow-up period compared to baseline. However, the rise in lactate in animals of the RIPC + HS group occurred later and was less prominent in the follow-up period compared to those of HS only group (Table 2, Fig. 4A). The timing of these events was synchronized with the partial recovery of tissue oxygenation.

**Table 2:** Acid base and oxygen delivery.

Variable	Control (C)			HS (H)			RIPC + HS (R)			*p* Value between groups		
	Base	EndBld	FU	Base	EndBld	FU	Base	EndBld	FU	Comparison	ΔBld	ΔFU
HCO_3_^−^ [mmol/lit]										C/H	< 0.001	0.004
	29.5 ± 3.6	30.3 ± 3.2†	31.2 ± 4.2	30.2 ± 1.9	27.3 ± 2.2†	27 ± 5.1†	28.9 ± 2.9	26.3 ± 2.8†	26.9 ± 1.4	C/R	0.001	0.038
				30.3 ± 2.3	27.9 ± 2.4†	29.9 ± 3.5	28.7 ± 3.1	25.4 ± 2.3†	27.4 ± 1.5	H/R	NS	NS
Lactate [mmol/lit]										C/H	0.001	0.012
	2.5 ± 0.8	2.5 ± 0.8	2.1 ± 1.1	2.2 ± 0.6	4.0 ± 1.7†	5.1 ± 4.0†	1.8 ± 0.4	3.1 ± 0.8	3.2 ± 1.5	C/R	0.002	0.039
				2.4 ± 0.3	3.6 ± 1.3	2.6 ± 1.0	1.8 ± 0.5	3.4 ± 0.7†	2.2 ± 1.0	H/R	NS	NS
pH										C/H	–^§^	–^§^
	7.46 ± 0.06	7.48 ± 0.05	7.46 ± 0.02	7.46 ± 0.07	7.42 ± 0.05†	7.39 ± 0.10	7.45 ± 0.05	7.43 ± 0.04	7.43 ± 0.03	C/R	–	–
				7.46 ± 0.08	7.43 ± 0.06	7.44 ± 0.05	7.43 ± 0.05	7.42 ± 0.05	7.43 ± 0.04	H/R	–	–
BE [mmolar]										C/H	< 0.001	0.011
	5.2 ± 2.1	6.1 ± 1.9†	6.5 ± 3.4	5.7 ± 2.8	2.4 ± 2.1†	1.5 ± 6.0	4.4 ± 2.7	1.9 ± 3.0†	2.3 ± 1.6	C/R	0.004	NS
				5.6 ± 3.2	3.1 ± 2.0	5 ± 3.3	3.7 ± 2.7	1.0 ± 2.8	2.6 ± 2.0	H/R	NS	NS
DO_2_ [mL/min]										C/H	< 0.001	< 0.001*
	458 ± 154	462 ± 142	459 ± 150	550 ± 73	234 ± 77†	299 ± 108†	500 ± 100	242 ± 64†	340 ± 114†‡	C/R	< 0.001	0.009*
				555 ± 63	258 ± 85†	348 ± 85†	504 ± 100	233 ± 77†	386 ± 117‡	H/R	NS	0.075*
ER										C/H	< 0.001	0.073*
	0.25 ± 0.06	0.28 ± 0.11	0.32 ± 0.18	0.23 ± 0.03	0.60 ± 0.13†	0.54 ± 0.16†	0.25 ± 0.04	0.57 ± 0.10†	0.44 ± 0.11†‡	C/R	< 0.001	0.005*
				0.22 ± 0.02	0.54 ± 0.13†	0.45 ± 0.10†	0.25 ± 0.02	0.61 ± 0.07†	0.43 ± 0.07†‡	H/R	NS	NS*
VSaO_2_ [%]										C/H	< 0.001	0.001
	80 ± 5	76 ± 11	71 ± 19	82 ± 3	43 ± 14†	48 ± 17†	81 ± 4	46 ± 11†	57 ± 10†	C/R	< 0.001	0.055
				84 ± 2	49 ± 15†	59 ± 11†	80 ± 2	42 ± 8†	61 ± 8†‡	H/R	NS	NS

Data presented as mean ± SD. Numbers in first row of each parameter are for all animals. Numbers in second row of each parameter are only those that survived (6 h of follow-up: Controls, n = 7 at all time-points; HS, n = 13 at 2H, n = 10 at 4H, n = 9 at 6H; RIPC + HS, n = 8 at 2H and 4H, n = 5 at 6H). Normality was determined by Shapiro–Wilk (SW) test (*p* > 0.1). Comparisons between baseline, end of bleeding [EndBld], and follow-up [FU] (mean over 7-h follow-up) within groups were performed by paired t-test with Bonferroni’s correction for multiple comparisons or Friedman’s test with pairwise comparisons as *post-hoc* test as appropriate (according to SW). Comparisons between groups (relates to all 7 h) were performed by analysis of variance (ANOVA) with Fisher’s least significance difference (FLSD) as *post-hoc* test or Kruskal–Wallis (KW) with Conover-Iman as *post-hoc* testHCO_3_^−^ = Bicarbonate, BE = Base Excess; DO_2_ = Delivered Oxygen, ER = Oxygen Extraction Ratio, VSaO_2_ = Mixed venous saturation. ΔBld = (EndBld-Baseline), ΔFU = (FU-EndBld), *ΔFU = (FU-Baseline). *p* < 0.05 considered significant and all tests 2-tailed. †*p* < 0.05 versus baseline; ‡*p* < 0.05 versus EndBld; ^§^not significant by ANOVA/KW.

Global Oxygen Delivery (DO_2_) was significantly greater during the follow-up period compared to the end of bleeding in animals of the RIPC + HS group (DO_2,_ RIPC + HS, Follow-up -vs- End of Bleed: 340 ± 114 -vs- 242 ± 64 ml/min, *p* = 0.04), but not in those of the HS only group (HS-only: 299 ± 108 -vs- 234 ± 77 ml/min, *p* = 0.12) (Table 2, Fig. 4B). This was the case whether the analysis was performed across all animals (above) or restricted to those that survived at 6 h of follow-up (RIPC + HS, *p* = 0.02; HS-only, *p* = 0.06) (Table 2, Fig. 3).

The Tissue Oxygen Extraction Ratio (ER) in animals of the RIPC + HS group was significantly lower during the follow-up period than at the end of bleeding (ER, RIPC + HS, Follow-up -vs- End of Bleed: 0.44 ± 0.11 -vs- 0.57 ± 0.10 ml/min, *p* = 0.028) but not in those of the HS only group (HS-only, 0.60 ± 0.13 -vs- 0.54 ± 0.16 ml/min, *p* = 0.427) (Table 2, Fig. 4C). This reduction in tissue oxygen extraction was found whether the analysis was performed across all animals (above) or restricted to those that survived at 6 h of follow-up (RIPC + HS, *p* = 0.01 and HS-only *p* = 0.11) with the ER levels in those of the RIPC + HS group approaching those of the control group (Table 2, Fig. 3).

### Renal function tests (Table 3)

**Table 3:** Kidney Functions.

Variable	Control (C)			HS (H)			RIPC + HS (R)			*p* Value between groups		
	Base	EndBld	FU	Base	EndBld	FU	Base	EndBld	FU	Comparison	ΔBld	ΔFU
Urea [mmol/lit] (Slope)		(0.55 ± 0.14)			(0.60 ± 0.17)			(0.62 ± 0.19)		C/H	0.022	–^§^
	3.5 ± 1.1	3.7 ± 0.9†	5.6 ± 0.9†‡	4.1 ± 1.3	4.7 ± 1.5†	6.6 ± 1.9†‡	3.1 ± 1.0	3.7 ± 0.9†	5.8 ± 1.3†‡	C/R	0.01	–
				4.2 ± 1.4	4.7 ± 1.5†	6.6 ± 2.0†‡	3.2 ± 1.2	3.8 ± 1.0†	5.9 ± 1.3†‡	H/R	NS	–
Creatinine [μmol/lit] (Slope)		(0.17 ± 0.09)			(0.37 ± 0.08) ‖			(0.40 ± 0.07) ‖		C/H	< 0.001	< 0.001
	126 ± 27	126 ± 31	158 ± 48†‡	120 ± 27	157 ± 28†	235 ± 39†‡	111 ± 14	149 ± 16†	239 ± 15†‡	C/R	< 0.001	< 0.001
				122 ± 33	158 ± 34†	229 ± 42†‡	105 ± 13	143 ± 12†	228 ± 18†‡	H/R	NS	NS
K^+^ [mmol/lit]										C/H	< 0.001	0.003*
	4.6 ± 0.5	4.7 ± 0.7	6 ± 1.3†‡	4.2 ± 0.3	5.3 ± 0.6†	7.1 ± 1.1†‡	4.2 ± 0.2	5.3 ± 0.5†	6.9 ± 0.8†‡	C/R	< 0.001	0.013*
				4.2 ± 0.3	5.1 ± 0.5†	6.5 ± 1.0†‡	4.3 ± 0.1	5.5 ± 0.4	6.4 ± 0.6	H/R	NS	NS*
Phosphate [mmol/lit]										C/H	< 0.001	0.008
	2.7 ± 0.4	2.7 ± 0.3	3.4 ± 0.4†‡	2.6 ± 0.4	3.1 ± 0.5†	4 ± 1.0†‡	2.6 ± 0.2	2.9 ± 0.2†	3.7 ± 0.4†‡	C/R	0.001	0.078
				2.5 ± 0.3	2.9 ± 0.3†	3.5 ± 0.6†‡	2.6 ± 0.3	3 ± 0.3†	3.5 ± 0.5†‡	H/R	NS	NS
Na^+^ [mmol/lit]										C/H	–^§^	–^§^
	140 ± 1	138 ± 3	138 ± 2	139 ± 2	137 ± 3	137 ± 2†	139 ± 4	138 ± 3	137 ± 4	C/R	–	–
				139 ± 2	136 ± 2	136 ± 1	140 ± 3	139 ± 3	138 ± 4	H/R	–	–
Cl^-^ [mmol/lit]										C/H	–^§^	–^§^
	99 ± 1	97 ± 2	97 ± 2	98 ± 2	97 ± 2	95 ± 1†‡	99 ± 2	99 ± 2	97 ± 2†‡	C/R	–	–
				98 ± 2	97 ± 2†	96 ± 1†‡	100 ± 2	100 ± 2	98 ± 2	H/R	–	–

Data presented as mean ± SD. Numbers in first row of each parameter are for all animals. Numbers in second row of each parameter are only those that survived (6 h of follow-up: Controls, n = 7 at all time-points; HS, n = 13 at 2H, n = 10 at 4H, n = 9 at 6H; RIPC + HS, n = 8 at 2H and 4H, n = 5 at 6H). Numbers in parentheses represent slope. Normality was determined by Shapiro–Wilk (SW) test (*p* > 0.1). Comparisons between baseline, end of bleeding [EndBld], and follow-up [FU] (mean over 7-h follow-up) within groups were performed by paired t-test with Bonferroni’s correction for multiple comparisons or Friedman’s test with pairwise comparisons as *post-hoc* test as appropriate (according to SW). Comparisons between groups or slope (change over follow-up time calculated individually by linear regression) were conducted by analysis of variance (ANOVA) with Fisher’s least significance difference (FLSD) as *post-hoc* test or Kruskal–Wallis (KW) with Conover-Iman as *post-hoc* test. ΔBld = (EndBld-Baseline), ΔFU = (FU-EndBld), *ΔFU = (FU-Baseline). *p* < 0.05 considered significant and all tests 2-tailed. †*p* < 0.05 versus baseline; ‡*p* < 0.05 versus EndBld; ^§^not significant by ANOVA/KW; ‖ *p* < 0.05 versus Control by ANOVA.

Evidence of acutely compromised renal function was observed in animals that underwent bleeding with or without prior RIPC. Serum Urea increased during the follow-up, and was not different between those bled with or without prior RIPC. Serum creatinine doubled in the animals that underwent bleeding compared to non-bled controls (*p* < 0.001) (Table 3). Evidence of compromised renal function was also apparent according to RIFLE criteria in animals that underwent bleeding compared to controls. This included doubling of creatinine level over baseline in 12 of 13 [92%] HS-only and in 8 of 8 [100%] RIPC + HS, but in only 1 of 7 [17%] Controls (*p* < 0.001 by Fisher’s exact test for 2 × 3 tables). In addition, RIFLE (Risk, Injury, and Failure; and Loss; and End-stage kidney)^23^ “at risk” urinary output (< 0.5 ml/kg/hour) occurred in 10 of 13 [77%] HS-only and 7 of 8 [88%] RIPC + HS, but in only 2 of 7 [29%] control (*p* = 0.053). A significant increase in serum K^+^ was detected in both groups of animals that underwent bleeding (with or without RIPC) compared to non-bled controls (Table 3).

### Liver function tests (Supplemental Table S2 and Supplemental Figure S2)

A sharp increase in the liver enzymes Lactic Dehydrogenase (LDH), Alanine aminotransferase (ALT), and Aspartate aminotransferase (AST) was observed in 5 of the 13 animals of the HS-only group throughout the follow-up period but in only 1 of the 8 RIPC + HS, and in 1 of 7 controls (Supplemental Figure S2). However, ALP and GGT did not deviate significantly from normal throughout the experiment. A sharp increase in glucose was seen in animals that underwent bleeding (Glucose > 8 mmol/L: Control, 2 of 7 [29%] -vs- HS, 11 of 13 [85%] -vs- RIPC + HS, 7 of 8 [88%], *p* = 0.017). This rise in glucose was followed by a gradual decline during the follow-up period (*p* = 0.003 over-the-slope) to final levels that were not significantly different from baseline (*p* > 0.25). CPK was significantly higher compared to baseline across all groups. There were no impairments or differences in prothrombin time (PT), partial thromboplastin time (PTT), or international normalized ratio (INR) between groups (Supplemental Table S3).

### Blood cell count (Supplemental Table S3)

White blood cells (WBC) increased during the follow-up period in both groups that were bled. The platelet count decreased throughout the experiment in all groups but remained within the physiological range.

## Discussion

In this study we tested the effects of Limb RIPC on a variety of hemodynamic and biochemical parameters, as well as on survival, in a large animal model of moderate, controlled hemorrhagic shock. Whole blood, plasma, and fluids were withheld in order to simulate an extreme field situation of several hours delay in evacuation to the site of definitive medical care. We found no difference in survival between animals of the RIPC + HS group and those of the HS-only group. Nevertheless, the former appeared to be in a better hemodynamic and oxygenation state than those of the latter including a significantly higher heart rate and increased MAP, as well as increased DO_2_ and decreased ER.

Pigs were chosen as a model of hemorrhagic shock because of the high correspondence with human cardiovascular and hemodynamic responses^24^ and for comparison with previous studies in which pigs were used to investigate the efficacy of RIPC in vivo albeit in different models^25–27^. The current study was designed to tests the effect of RIPC in a large animal model of hemorrhagic shock without fluid return or replacement therapy. The goal was to assess the capabilities of RIPC by itself to prevent or slow the rapid deterioration of casualties with major blood loss in circumstances of delayed evacuation. We applied one of the possible RIPC protocols immediately before inducing hemorrhage with the understanding that this could be optimized in future studies or abandoned in favor of pharmacological intervention after identifying and characterizing the relevant putative circulating factor(s) that mediate(s) the desired effects.

We found no difference in overall mortality between animals of the RIPC + HS group and those of the HS-only group. This is in contrast with the study of Hu et al. who used a similar RIPC protocol in a rat model and reported significantly improved survival after 72 h with improved ejection fraction, myocardial performance index, sublingual microvascular flow index, and neurological deficit score. In their study, approximately 50% of the total blood volume was removed over an hour with resuscitation including return of extracted blood beginning 30 min later and completed within the next 30 min^19^. Of interest, these myocardial and cerebral protective effects of RIPC were inhibited by the K-ATP channel blocker, glibenclamide.

Dai et al.^20^ reported improved survival over the longer term (6 weeks) in rats subjected to withdrawal of blood to a fixed mean blood pressure of 30 mmHg, maintaining this value for 30 min, and then reinfusing the withdrawn blood (#animals survived / total: RIPC vs no RIPC: 13/26 (50%) vs 5/27 (19%), *p* = 0.02). Despite the lack of positive effect on survival in the current swine study, as indicated above, animals of the RIPC + HS group had significant improvements in several important hemodynamic parameters. Animals of the RIPC + HS group maintained a significantly higher heart rate as well as increased MAP (Fig. 2) following bleeding and during the entire seven hours of follow-up compared to the other groups. This is of added significance in view of the fact that these animals received no fluids or replacement therapy pointing to the possibility that RIPC might increase the nature and duration of resilience to such an injury by improving key parameters of physiological competence.

Animals in the RIPC + HS group had less decrease in pH and bicarbonate compared with the HS only group, and the increase in lactate began at a later time point. Decreased lactate has also been reported in other RIPC models^17,28^. These findings are another important biochemical manifestation of the attenuating effect of RIPC on the consequences of major hemorrhage.

The increase in DO_2_ and decrease in ER found in the current study in animals of the RIPC + HS group point to an improved cellular response and better recovery of tissue oxygenation after the ischemic insult, and its many potential consequences, caused by the hemorrhage (Table 2, Fig. 4B,C).

The compromise in renal function was apparent by multiple criteria in both groups of animals with HS–with or without RIPC. This is not surprising in view of the fact that these animals were bled without fluid resuscitation. RIPC did not appear to have any protective advantage for kidney function, and elevated potassium seen in both groups is of great concern also because of its high arrhythmogenic potential. Further studies are in order to determine the differential efficacy of adding appropriate fluid replacement to the RIPC protocol or adding other pre-conditioning interventions, or combinations thereof, prior to hemorrhage.

The rise in the hepatocellular enzymes LDH, ALT and AST seen primarily in the HS-only group is consistent with what occurs commonly in shock and its associated arterial ischemia. It is also common, as was found here, that the enzymes ALP and GGT remain normal, particularly early on in shock, since these are known to be less sensitive to ischemia but more sensitive to congestion and obstruction. Although the precise explanation for why 7 of 8 animals of the RIPC + HS group were spared the rise in hepatocellular enzymes remains to be investigated, its positive effects on tissue oxygenation (increased tissue DO_2_ and decreased ER) found in this study would appear to be prominent manifestations of this effect.

The sharp rise in glucose level, observed in 85% of the animals that underwent bleeding, was most probably a component of the stress response. This is consistent with the elevation in cortisol levels relative to baseline observed at the end of bleeding (Supplemental Table S3). We did not find any significant changes in coagulation tests from baseline control values across all groups. This might be due to lack of significant tissue trauma or because the follow-up period was limited to 7-h.

As expected, we found an increase in mean neutrophils in all groups in the follow-up period. This is a recognized component of the general stress response. It has been suggested that there may be a role for neutrophils in tissue-protective properties of RIPC^29^. In view of the wide range of functions and responses of neutrophils in circumstances of traumatic injury and stress, further studies dedicated to this issue are warranted to determine whether the early increase in WBC seen in the RIPC group is of positive or negative effect in this model^29^.

We observed some differences in total platelets, but all were within normal range. Platelet dysfunction, sometimes found in exsanguinating victims, can contribute to trauma-induced coagulopathy^30,31^. Further studies are warranted in order to determine the effect of RIPC on platelet function and coagulation in this model over more prolonged follow-up periods.

The customary anesthesia protocol for large animal studies in our institution includes induction with propofol and maintenance with isoflurane. Although RIPC has been shown to be an effective adjunctive cardioprotective strategy in many small randomized clinical trials (RCTs) across multiple surgical procedural settings, two, large, multicenter RCTs demonstrated no obvious improvement in clinical outcomes following RIPC^14,15^. This was attributed to the use of propofol that was reported to attenuate RIPC-induced cardioprotective effects^32,33^. In the study by Meybohm^15^, upper-limb RIPC, performed while patients were under propofol-induced anesthesia, did not show a relevant benefit among patients undergoing elective cardiac surgery. However, since it appears that all patients in that study were given propofol, it is difficult to determine with certainty whether propofol was responsible for the lack of benefit of RIPC in that study.

The exact mechanism underlying possible RIPC-induced cardioprotective effects is not known. However, it was suggested that RIPC increases endogenous plasma exosomes or microvesicles (possibly platelet-derived)^34^ that can deliver signals to the myocardium and other tissues to provide protection against ischemia and reperfusion injury by a pathway involving TLR4 and protective heat shock proteins^34–37^. Of interest, Abel et al. reported that extracellular microvesicles isolated from patients undergoing RIPC versus sham were associated with decreased hypoxia-evoked apoptosis of cardiomyoblasts after isoflurane – but not after propofol^15,38^. Yu et al.^39^, in a rodent model, suggested that propofol blunts the cardioprotective effect of RIPC through its effect on the cardiac TRPV1 channel only if it is administered before RIPC, but not when propofol is given after RIPC^40^. Thus, the effect of propofol on RIPC remains an unresolved issue.

Looking at translational perspectives of the study findings, RIPC as a prophylactic intervention in the form of an inflated cuff around the arm might improve preparedness for a future potential planned encounter. However, the appropriate timing of such an intervention has yet to be resolved, and application of an occlusive tourniquet the night before a mission may be of questionable practicality. Moreover, traumatic injury usually does not occur with a fixed, pre-planned schedule. Hence, further studies designed to identify and characterize the endogenous systemic mediators of the effects of such remote preconditioning^10,41–45^ should provide the basis for the development of novel therapeutic agents that can be administered prophylactically, or after the fact, improving function, and possibly survival, in situations of life-threatening hemorrhage. Such strategies also might be of benefit for conservation or improvement of essential hemodynamic and metabolic functions in the setting of elective surgery. The results of the current study suggest that RIPC, by improving certain key parameters of physiological competence, might improve resilience to hemorrhage and hence slow the rapid deterioration of trauma casualties and extend the “golden hour” in circumstances of prolonged field care and delayed evacuation.

### Limitations

Since there are no previous studies using RIPC in a situation of hemorrhagic shock without fluid replacement on which to rely, the power analysis was based on extrapolation from other models. The number of pigs in the current study turned out to be not sufficient to reach significant statistical separation regarding effect of RIPC on survival. Nonetheless, this study was sufficiently powered to identify significant differences in certain essential hemodynamic and metabolic parameters and tissue oxygen delivery that point to potential benefit for attenuating the consequences of hemorrhage- induced ischemia an improving resilience. In view of the requirements of the ethics committee, including heavy anesthesia and analgesia as part of most animal models, our model did not reflect the situation of overstimulated sympathetic nervous system prior to hemorrhagic shock, although it is a known component in situations before emergency transport such as the traumatic hemorrhage during combat. However, the current model does reflect the situation of hemorrhagic shock without fluid replacement.

To conclude, the improvements in animals with prior RIPC (RIPC + HS) in hemodynamic and metabolic status, with better recovery of tissue oxygenation, provide essential substrates for improved cellular responses after hemorrhage and reduction of the likelihood of potentially catastrophic consequences of the accompanying ischemia. Many complexities have been encountered in the evaluation of the effects of RIPC in these and studies by others. Nonetheless, evidence of improvements in several important physiological parameters dictates that resolution of the question of efficacy of RIPC, and its putative circulating mediators, for improvement of resilience and prolongation of survival after hemorrhage, should not be abandoned. This is of particular importance in military or civilian situations where every minute counts, where fluid replacement or “scoop-and-run” are not possible due to tactical constraints, and where transport to a treatment center for definitive care is delayed.

## Methods

This study conformed to the Guide for the Care and Use of Laboratory Animals, (National Academy Press, Washington, D.C. 1996). Animal care and procedures were approved by the Ethics Committee of The Faculty of Medicine of The Hebrew University of Jerusalem (MD-13-13751-3). All methods are reported in accordance with ARRIVE guidelines^46^.

### Study design

Twenty-eight, domestic female pigs (Laboratory Animal Farm, Lahav, Israel, 47.2 ± 5.7 kg, age 4 months) were assigned to 3 groups (Fig. 1A): (1) C = control, no procedure(n = 7); (2) HS = hemorrhagic shock (35% of total blood volume) (n = 13); (3) RIPC + HS = remote ischemic preconditioning (3 cycles of 5' occlusion/reperfusion followed by HS (RIPC, Fig. 1B) (n = 8). Four additional animals were subjected to RIPC alone as required by the ethics committee for validation of the safety of the procedure. After completion of the initial procedures, all animals were observed for 7 h (or until death if occurred earlier) with no fluid or replacement therapy. Hemodynamic parameters were documented every 5 min until the end of bleeding and then every 20 min during the observation period. Blood and urine were collected at baseline, at the end of the RIPC, at the end of bleeding, and every hour during the observation period. Animals that survived until the end of the observation period were euthanized.

### Surgical protocol

#### Preparation and anesthesia

Animals were sedated with xylazine (1 mg/Kg, IM). Anesthesia was induced with ketamine (10 mg/Kg, IM). Animals were given a mixture of diazepam (2 mg, IV), ketamine (400 mg, IV), and propofol (1–4 mg/Kg, IV). Tramadol (5 mg/Kg, IM) was administered for analgesia. Cefazolin (1 g, IV) was given prophylactically. The pigs were intubated with a 7.0 mm cuffed silastic endotracheal tube. Anesthesia was maintained with 2% isoflurane in 100% oxygen, and animals were ventilated using controlled mechanical ventilation. Tidal volume was set to 10 mL/kg with respiratory rate of 13–15 breaths/min. to reach an end tidal CO_2_ of 35 mmHg at baseline.

The left common carotid artery was cannulated by the over-the-wire Seldinger technique for monitoring of blood pressure and heart rate. The pulmonary artery catheter (Swan-Ganz CCOmbo, Edwards Lifesciences, Irvine, CA) was inserted via the internal jugular vein. The right femoral artery was cannulated for arterial blood sampling and withdrawal of blood during the controlled hemorrhage.

The left femoral artery was surgically exposed for clamping to perform the ischemic pre-conditioning procedure. A urinary catheter was inserted for output monitoring. Body temperature was monitored rectally. A pulse oximeter was placed on the tongue or tail to measure oxygen saturation. Continuous three-lead ECG was monitored using electrodes placed on the right forelimb, left forelimb, and left hind limb. At the end of the post-procedural observation period, surviving animals were euthanized by intravenous injection of KCl.

#### Remote ischemic preconditioning

The exposed femoral artery was subjected to 3 sequential cycles of 5 min occlusion using a Dietrich Bulldog Artery Clamp (19-8099, Codman, Raynham, MA) followed by 5 min of reperfusion according to the method described by Zhang et al.^47^. Angiography was performed to confirm occlusion of blood flow (Fig. 1B). We chose this surgical occlusion technique following pilot experiments that showed it was not possible to reliably maintain adequate ischemia by using external compression on the hind legs.

#### Hemorrhagic shock

The blood volume was calculated considering individual animal weight according to the conversion formula 67.3 ml/kg^48^. Hemorrhage was simulated by withdrawing approximately one-third of the animal’s calculated blood volume^49^ in 50 ml aliquots. The rate of bleeding was controlled to keep MAP from dropping below 30 mmHg. When necessary, bleeding was stopped and the animal allowed to recover prior to resumption of bleeding. The total bleeding time was up to 60 min with the exception of 2 animals (75 and 80 min) in which MAP decreased to a dangerously low value and additional time was given to let the animals recover. A sensitivity analysis was conducted to determine if these 2 animals biased the results. No major differences were found when comparing the results with or without them (See detailed results of the sensitivity analysis in Supplemental Table S4).

### Hemodynamic parameters

Systolic blood pressure (SBP), diastolic blood pressure (DBP), MAP, and HR were monitored continuously using a Datex-Ohmeda Cardiocap 5 (Datex-Ohmeda Inc, Madison, WI). Cardiac output (CO) was continuously monitored using a Vigilance II Monitor (Edwards Lifesciences, Irvine, CA). Measurements were documented manually every 5 min until the end of bleeding, and then every 20 min during the observation period.

### Blood/urine sample analysis

Blood samples were analyzed for arterial and mixed venous blood gasses (pO_2_, pCO_2_, pH, HCO_3_^−^, O_2_ saturation and BE) using the Cobas-b-221 blood gas system (Roche Diagnostics, Indianapolis, IN). Lactate was analyzed by Stat Strip Xpress (Nova Biomedical Corporation, Waltham, MA). Samples for complete blood count, coagulation tests, blood chemistry (electrolytes, CPK, liver enzymes [Bilirubin, AST, ALT, ALP, GGT], renal function tests [Urea, Creatinine], and glucose), and total serum cortisol were analyzed by standard clinical laboratory analysis. Urine was collected at each time point throughout the experiment.

### Tissue oxygenation

Oxygen Delivery (DO_2_) was calculated as: DO_2_ = CaO_2 _× CO, where CaO_2_ is the concentration of oxygen in hemoglobin plus its concentration dissolved in plasma multiplied by CO as described previously^22^. Oxygen Extraction Ratio (ER) from tissues was calculated as: ER = VO_2_ / DO_2_, where VO_2_, the oxygen consumption, = arterial oxygen content minus mixed venous oxygen content (CaO_2_–CvO_2_) × CO^22^.

### Data analysis

Time points for collection of data from baseline through the end of the 7-h follow-up are indicated in Fig. 1. To synchronize the analysis of the hemodynamic data with the rest of the data, the last hemodynamic measurement of each procedure represented the value at the end of the procedure (RIPC or bleeding), and the average of the 3 data points during each follow-up hour represented that hour. The amount of urine collected was summed individually for each animal over all time points (excluding the first residual) and divided by weight and survival time to achieve urine output rate [ml/kg/h]. The acute response to RIPC and/or bleeding for each variable of each animal was reported as the difference from baseline or from the end of bleeding (ΔRIPC [EndRIPC minus– Baseline] or ΔBld [EndBld minus– Baseline]). The long-term response was calculated by averaging each variable of each animal over the 7-h follow-up period and subtracting it from the individual baseline or end of bleeding value. For variables that were characterized by a steady increase or decrease throughout the follow-up period (Creatinine [CRE], Urea, glucose [GLU], Potassium [K^+^], Phosphate [P]), the change over time (slope) was calculated using linear regression. RIFLE criteria were also used to assess kidney function.

Analyses restricted to those animals that survived were performed at the 6 (and not 7)-hour follow-up time point where there were at least 5 animals that survived in each group.

### Statistical methods

Sample size: In the study of Chudnofsky et al.^50^, the overall survival of young swine that were bled at a rate of 1.25 mL/kg/min, and to which fluid was returned after 45 min, was 57%. In the current study, the bleeding was less severe, and the pigs fully grown, but fluids were not returned for 7 h. We therefore expected an overall survival of no more than 20% during the long follow-up period despite the known resilience of pigs. In the absence of previous pig studies of RIPC prior to hemorrhagic shock, we based our expected overall survival rate after RIPC on the study by Hu et al. in rats^19^ that showed that an RIPC regimen, similar to ours, prior to severe hemorrhagic shock, but with resuscitation, improved overall survival from 19 to 100% with RIPC. Since our model was without fluid replacement, we assumed that the RIPC-associated benefit on survival will be reduced to 70%. With these assumptions, 10 animals per group were calculated to be sufficient to achieve an 83% power at significance level of 0.05. Sample size was determined with PASS software (NCSS Statistical Software, Kaysville, UT).

Continuous variables were reported as mean ± SD and by 95% confidence intervals (Lower Confidence Interval = LCI, Upper Confidence Interval = UCI) where required. Categorical variables were reported as counts and percentages. Normality of distribution was based on Shapiro–Wilk (SW) test (*p* > 0.1). Within-group comparisons between 2 time points for acute RIPC effects (baseline, EndRIPC) were performed with paired t-test/Wilcoxon (according to the SW test). Within-group for 3 time points (baseline, EndBld, FU) were performed with repeated-measures ANOVA using Bonferroni’s correction for multiple comparisons or Friedman’s test with pairwise comparisons as *post-hoc* test as appropriate. Between-group comparisons of ΔRIPC = (EndRIPC-Baseline), ΔBld = (EndBld-Baseline), ΔFU = (FU-EndBld) or *ΔFU = (FU-Baseline) for 2 groups were conducted with 2-sample t-test (with a preceding Leven's test for equal variances when necessary) or Mann–Whitney-U(MWU) test (according to the SW test). When comparing more than 2 groups, we used analysis of variance (ANOVA) or Kruskal–Wallis (KW) with Fisher’s least significance difference (FLSD) or Conover-Iman as *post-hoc* tests, respectively. Categorical results were tabulated in contingency tables and significance determined with the Chi-square test or Fisher’s exact test as appropriate. Survival analysis was performed with Kaplan–Meier statistics. *p* < 0.05 Was considered significant, and all tests were 2-tailed except for the 1-tail 2X3 Fisher’s exact test. Statistical analyses were performed with SYSTAT, version 13 (Systat Software, Chicago, USA).

### Ethical approval and informed consent

Ethics approval # MD-13-13751-3 was given by the Ethics Committee of The Faculty of Medicine of The Hebrew University of Jerusalem.

## Supplementary Information

Supplementary Information.

## Data Availability

Requests for additional details regarding the data should be addressed to the corresponding author.

## Abbreviations

ALP: Alkaline phosphatase
ALT: Alanine aminotransferase
AST: Aspartate aminotransferase
BE: Base excess
CI: Cardiac index
CO: Cardiac output
CPK: Creatine phosphokinase
DBP: Diastolic blood pressure
DO_2_: Global oxygen delivery
ER: Tissue oxygen extraction ratio
FU: Follow-up period
GGT: γ-Glutamyl transferase
HCO_3_: Bicarbonate
HR: Heart rate
HS: Hemorrhagic shock
INR: International normalized ratio
LCI: Lower Confidence Interval
LDH: Lactic dehydrogenase
MAP: Mean arterial pressure
MST: Mean survival time
PCWP: Pulmonary capillary wedge pressure
PT: Prothrombin time
PTT: Partial thromboplastin time
RCTs: Randomized clinical trials
RIFLE: Risk, injury, and failure; and loss; and end-stage kidney
RIPC: Remote ischemic preconditioning
SV: Stroke volume
SBP: Systolic blood pressure
UCI: Upper confidence interval
VSaO_2_: Mixed venous saturation
WBC: White blood cells
