# Supplementary material for: Remote ischemic preconditioning improves tissue oxygenation in a porcine model of controlled hemorrhage without fluid resuscitation
Source: Sci Rep. 2021 May 24;11:10808. doi: 10.1038/s41598-021-90470-6 (PMC8144617; doi:10.1038/s41598-021-90470-6)
Supplement: Supplementary file 1 — Supplementary Information. [file 41598_2021_90470_MOESM1_ESM.pdf]

Supplemental Fig. S1: Survival

Kaplan-Meier Survival curve showing no difference between RIPC+HS and HS alone (p=0.471 by log rank test by the Breslow-Gehan method). #animals: Controls n=7; HS n=13; RIPC+HS n=8.

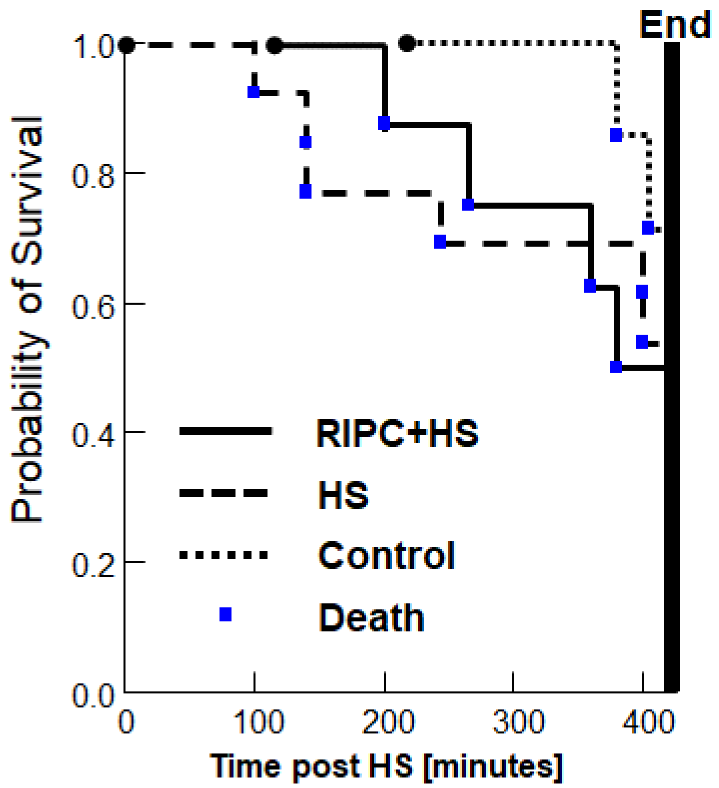

Supplemental Fig. S2: Liver Enzymes

Individual measurements of Lactic Dehydrogenase (LDH), Alanine aminotransferase (ALT), and Aspartate aminotransferase (AST). Each line represents an individual animal of each group (HS [red], RIPC+HS [blue], Control [black]). Grey zone shows time of bleeding. Note that 5 or 6 of 13 animals from the HS group (bleeding only) had a sharp increase in the liver enzymes suggesting hepatocellular damage that was mostly mitigated or delayed by RIPC. R=time of RIPC, Bl=time of bleeding. #animals: Controls n=7; HS n=13; RIPC+HS n=8

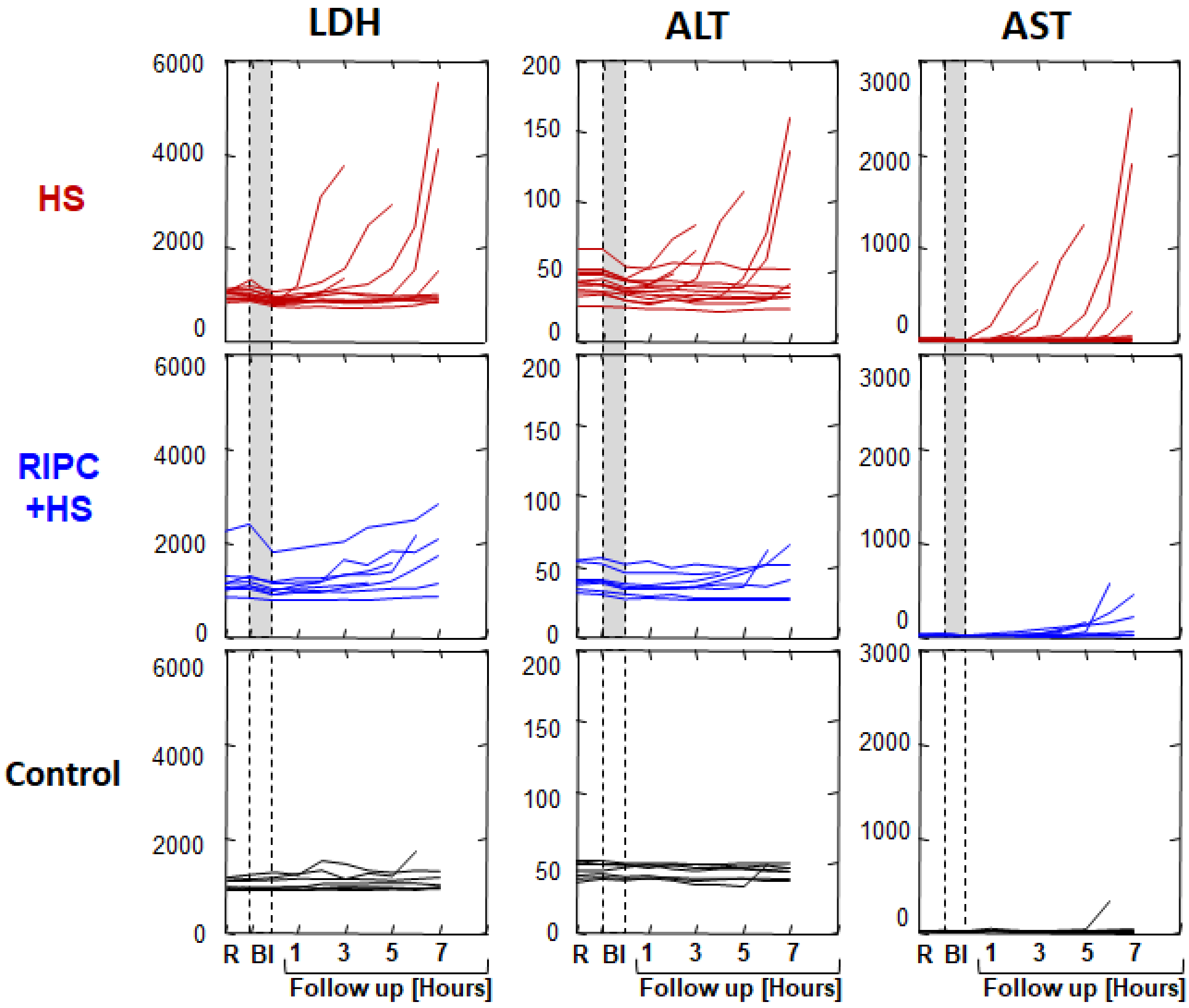

Table S1: RIPC Acute Effects

Category	Variable	Without RIPC (n=20)			With RIPC (n=12)			p-value - RIPC: with vs without	
		Base	EndRIPC	p-value	Base	EndRIPC	p-value	Base	ΔRIPC
Hemodynamics	MAP [mmHg]	66±10	66±10	0.112	66±8	63±7	0.867	0.99	0.33
	HR [bpm]	85±11	80±10	0.003	81±14	75±10	0.008	0.43	0.76
	CI [l/min/m^2]	3.7±0.7	3.6±0.7	1.00	3.8±1.2	3.6±1.3	0.468	0.70	0.39
	SV [ml]	40.4±8.6	43.1±10.1	0.007	43.2±14.8	43.3±15.5	0.937	0.55	0.27
	PCWP [mmHg]	8.6±4.8	8.7±4.1	0.915	10.4±5.1	10.3±5.9	0.774	0.32	0.84
Blood Gas	HCO_3^- [mmol/lit]	30.0±2.6	30.1±2.4	0.44	28.8±3.0	28.8±2.8	0.83	0.25	0.72
	Lactate [mmol/lit]	2.3±0.7	2.6±1.4	0.41	1.9±0.8	2.0±0.8	0.54	0.024	0.90
	pH	7.46±0.07	7.47±0.06	0.42	7.45±0.04	7.46±0.05	0.11	0.38	0.30
	BE [mmolar]	5.5±2.5	5.7±2.3	0.23	4.2±2.7	4.5±3.0	0.14	0.18	0.69
	$VSaO_2$ [%]	82±4	80±6	0.11	80±6	80±6	0.94	0.44	0.30
Oxygen Delivery	DO_2 [mL/min]	518±114	522±126	0.78	528±173	494±199	0.36	0.85	0.30
	ER	0.24±0.04	0.25±0.06	0.15	0.25±0.05	0.26±0.06	0.88	0.25	0.28
Kidney Function	Urea [mmol/lit]	3.9±1.2	4.0±1.2	0.27	3.1±1.0	3.2±0.9	0.33	0.072	1.0
	Creatinine [µmol/lit]	122±26	121±28	0.43	115±14	115±15	0.99	0.35	0.82
Serum Electrolytes	K^+ [mmol/lit]	4.4±0.4	4.5±0.4	0.001	4.3±0.4	4.6±0.4	0.001	0.68	0.013
	Phosphate [mmol/lit]	2.7±0.4	2.7±0.3	0.035	2.7±0.3	2.7±0.3	0.056	0.96	0.43
	Na^+ [mmol/lit]	139±2	139±2	0.88	139±3	138±4	0.46	0.67	0.6
	Cl^- [mmol/lit]	98.1±1.5	98.0±2.0	0.56	98.8±1.9	97.8±2.4	0.007	0.26	0.041
Liver Function	Glucose [mmol/lit]	6.3±1.7	6.6±1.6	0.043	6.1±1.9	6.8±2.2	0.028	0.75	0.21
	ALP [U/lit]	146±29	147±30	0.41	149±59	150±58	0.37	0.89	0.80
	GGT [U/lit]	46.5±17.0	49.8±20.3	0.17	36.0±7.6	36.2±6.4	0.82	0.023	0.21
	LDH [U/lit]	1012±98	1028±128	0.48	1168±355	1195±393	0.34	0.13	0.77
	AST [U/lit]	24.5±4.9	24.4±5.9	0.91	28.8±6.8	28.9±8.4	0.92	0.068	0.89
	ALT [U/lit]	43.7±8.8	43.7±8.6	0.83	39.3±7.9	39.8±8.2	0.40	0.17	0.47
	CPK [U/lit]	959±432	1030±483	0.009	878±359	971±433	0.003	0.57	0.49
Coagulation	PPT [sec]	15.8±1.9	16.5±2.3	0.11	14.4±1.9	14.7±1.5	0.45	0.024	0.58
	PT%	137±18	139±16	0.49	147±22	153±24	0.028	0.15	0.55
	INR	0.84±0.04	0.84±0.03	0.81	0.83±0.05	0.82±0.06	0.024	0.47	0.15
	Platelets	340±100	332±108	0.26	329±120	299±125	0.09	0.78	0.17

Blood Count	HGB [gr%]	10.4±1.0	10.4±1.0	0.74	10.5±0.5	10.5±0.6	0.55	0.76	0.46
	HCT [%]	30.6±2.9	30.4±3.0	0.67	31.0±1.8	30.9±1.7	0.58	0.66	0.92
	RBC [10^{12}/lit]	6.2±0.5	6.2±0.5	0.99	6.3±0.3	6.3±0.4	0.71	0.43	0.77
	WBC [10^{9}/lit]	19.1±7.8	18.3±8.1	0.23	17.0±7.2	19.3±8.2	0.77	0.45	0.032
	Neutrophils [10^{9}/lit]	3.6±6.2	2.9±7.0	0.74	5.0±6.6	6.9±7.8	0.25	0.57	0.28
	Neutrophils %	16.5±23.6	11.3±23.7	0.58	27.7±29.1	33.8±33.8	0.45	0.25	0.29
	Lymphocytes [10^{9}/lit]	14.8±7.9	15.1±7.5	0.93	12.1±7.0	12.2±9.2	0.98	0.33	0.96
	Lymphocytes %	78.2±25.8	82.9±25.7	0.53	64.9±31.6	65.6±33.8	0.95	0.23	0.76

Data presented as mean±SD. Normality was determined by Shapiro-Wilk (SW) test ($p>0.1$). Comparison between baseline (Base) and end of RIPC (EndRIPC) within groups were performed by paired t-test or Wilcoxon Sign Rank test (according to SW). Comparisons between groups were performed by 2-sample t-test or Mann-Whitney-U test (according to SW). MAP=mean arterial pressure; HR=heart rate; CI=cardiac index; SV=stroke volume; PCWP=Pulmonary capillary wedge pressure; HCO_3^-=Bicarbonate; BE=Base Excess; $VSaO_2$=Mixed venous saturation; DO_2=Delivered Oxygen; ER=Oxygen Extraction Ratio; ALP=Alkaline Phosphatase; GGT=γ-glutamyltransferase; LDH= Lactic Dehydrogenase; AST=Aspartate aminotransferase; ALT=Alanine aminotransferase; CPK=Creatine phosphokinase; PPT= partial thromboplastin time; PT= prothrombin time; INR= international normalized ratio; HGB=hemoglobin; HCT=hematocrit; RBC=red blood cells; WBC=white blood cells.. ΔRIPC=(EndRIPC-Baseline). $p<0.05$ considered significant and all tests 2-tailed.

Supplemental Table S2: Liver Functions

Variable	Control (C)			HS (H)			RIPC+HS (R)			p-value between groups		
	**Base**	**EndBld**	**FU**	**Base**	**EndBld**	**FU**	**Base**	**EndBld**	**FU**	**Comparison**	**ΔBld**	**ΔFU**
Glucose		(-0.02±0.25)			(-1.20±1.10)			(-0.88±0.68)		C/H	-§	<0.001
[mmol/lit]	6.0±1.0	7.0±1.5†	7.7±1.6	6.4±1.9	10.3±3.1†	7.4±3.3‡	6.4±1.7	9.8±3.6†	7.2±3.0‡	C/R	-	0.002
(Slope)				6.5±2.2	10.7±2.8†	8.9±2.7†‡	6.5±1.7	10.2±4.7	8.3±3.7	H/R	-	NS
ALP										C/H	0.001	-§
[U/lit]	130±34	130±33	144±39	155±23	143±22†	169±33‡	167±58	155±55†	188±72‡	C/R	0.004	-
				156±24	140±21†	158±29‡	149±61	142±62	169±84	H/R	NS	-
GGT										C/H	-§	0.003
[U/lit]	41.7±13.4	51.0±21.8	54.0±24.5†‡	49.1±18.6	51.2±27.3	50.3±20.5	35.6±8.5	33.3±6.3	34.5±7.2	C/R	-	0.002
				52.8±19.8	57.1±30.2	52.9±22.3	37.4±9.9	35.2±7.3	36.1±8.3	H/R	-	NS
LDH										C/H	<0.001	-§
[U/lit]	1036±113	1054±145	1101±182†‡	999±91	895±86†	1263±583‡	1228±430	1209±314†	1354±446‡	C/R	0.001	-
				979±87	873±74†	959±193	1247±564	1080±411†	1324±542‡	H/R	NS	-
AST										C/H	0.017	0.009
[U/lit]	25.4±4.0	23.7±4.7	31.3±26.3	23.9±5.4	19.9±5.5†	176.4±213.2‡	27.6±6.2	22.9±5.4†	72.5±55.6‡	C/R	0.005	0.028
				24±5.7	19.2±5.7†	50.3±67.7‡	25.6±6.9	21±6.0†	51.4±42.3‡	H/R	NS	NS
ALT										C/H	<0.001	-§
[U/lit]	44.4±6.1	44.0±5.2	43.3±5.0	43.2±10.2	37.8±7.8†	45.4±14.3	41.4±8.4	37.3±8.1†	39.2±8.4	C/R	0.005	-
				41.2±11.4	36±8.5†	36.7±9.2	39.6±9.1	35.6±9.6†	36.4±9.7	H/R	NS	-
										C/H	-§	-§
CPK [U/lit]	909±531	972±553	1084±696	986±391	1022±466	1306±682‡	810±242	884±320†	1291±520†‡	C/R	-	-
				978±398	958±426	1189±668	674±185	724±292	1069±568	H/R	-	-

Data presented as mean±SD. Numbers in first row of each parameter are for all animals. Numbers in second row of each parameter are only those that survived (6 hours of follow-up: Controls, n=7 at all time-points; HS, n=13 at 2H, n=10 at 4H, n=9 at 6H; RIPC+HS, n=8 at 2H and 4H, n=5 at 6H). Numbers in parentheses represent slope. Normality was determined by Shapiro-Wilk (SW) test (p>0.1). Comparisons between baseline, end of bleeding [EndBld], and follow-up [FU] (mean over 7-hour follow-up) within groups were performed by paired t-test with Bonferroni's correction for multiple comparisons or Friedman's test with pairwise comparisons as *post-hoc* test as appropriate (according to SW). Comparisons between groups or slope (change over follow-up time calculated individually by linear regression) were conducted by analysis of variance (ANOVA) with Fisher's least significance difference (FLSD) as *post-hoc* test or Kruskal-Wallis (KW) with Conover-Iman as *post-hoc* test. ALP=Alkaline Phosphatase; GGT=γ-glutamyl transferase; LDH= Lactic Dehydrogenase; AST=Aspartate aminotransferase; ALT=Alanine aminotransferase; CPK=Creatine phosphokinase; ΔBld=(EndBld-Baseline), ΔFU=(FU-EndBld), *ΔFU=(FU-Baseline). p<0.05 considered significant and all tests 2-tailed. †p<0.05 vs baseline; ‡p<0.05 vs EndBld; §not significant by ANOVA/KW; ‖ p<0.05 vs Control by ANOVA.

Supplemental Table S3: Coagulation, Cortisol, and Blood Count Across Groups

Variable	Control (C)			HS (H)			RIPC+HS (R)			p value between groups		
	Base	EndBld	FU	Base	EndBld	FU	Base	EndBld	FU	Comparison	ΔBld	ΔFU
PPT [sec]										C/H	-§	-§
	15.8±2.1	16.4±1.7	15.8±2.6	15.8±1.9	14.0±2.5	16.8±2.8	14.0±1.1	13.4±1.3	14.8±1.1	C/R	-	-
										H/R	-	-
PT%										C/H	-§	-§
	131±19	140±14	145±17	139±17	150±24	135±14	156±21	155±32	157±22	C/R	-	-
										H/R	-	-
INR										C/H	-§	-§
	0.85±0.05	0.84±0.04	0.83±0.05	0.84±0.04	0.83±0.05	0.86±0.03	0.81±0.05	0.80±0.06	0.81±0.05	C/R	-	-
										H/R	-	-
Platelets										C/H	-§	-§
	301±49	276±67	262±49†	361±116	338±86	293±94†‡	336±88	319±69	254±66†‡	C/R	-	-
										H/R	-	-
Cortisol [U/lit]										C/H	0.009	0.007*
	373±186	428±170†	463±206	212±157	487±141†	505±141†‡	315±109	536±136†	553±156†‡	C/R	0.052	0.062*
										H/R	NS	NS*
HGB [gr%]										C/H	0.011	-§
	10.2±1.2	10.0±1.2	10.8±1.5‡	10.5±0.8	9.8±0.8†	11±.0±1.1‡	10.3±0.4	9.7±0.9	10.8±0.8‡	C/R	0.081	-
										H/R	NS	-
HCT [%]										C/H	0.007	-§
	29.8±3.7	29.4±4.0	32.0±4.8†‡	31.1±2.4	28.9±2.2†	33.0±3.3†‡	30.1±1.4	28.8±2.6	32.3±2.4†‡	C/R	NS	-
										H/R	NS	-
RBC [10^{12}/lit]										C/H	0.005	-§
	6.1±0.6	6.1±0.7	6.6±0.8‡	6.3±0.5	5.8±0.4†	6.5±0.6‡	6.2±0.3	5.9±0.6	6.6±0.5‡	C/R	NS	-
										H/R	NS	-
WBC [10^{9}/lit]										C/H	0.057	-§
	16.9±6.8	16.4±8.4	20.3±7.4	20.3±8.3	16.4±7.8†	27.2±7.6†‡	13.6±4.7	15.4±4.8	23.9±5.9†‡	C/R	NS	-
										H/R	0.002	-
Neutrophils [10^{9}/lit](%)										C/H	-§	-§
	3.7±6.8	4.4±6.6	6.9±6.8	3.5±6.1	3.2±4.3	8.3±5.3†‡	3.2±3.6	4.7±4.2†	10.3±4.3†‡	C/R	-	-
	(17±27%)	(27±33%)	(31±22%)	(16±23%)	(22±26%)	(29±17%)	(27±28%)	(31±27%)	(42±12%)	H/R	-	-
Lymphocytes [10^{9}/lit](%)										C/H	-§	-§
	11.2±7.0	11.9±9.1	12.7±6.5	16.7±8.0	13.1±8.5	18.6±6.7‡	10.3±6.4	10.5±4.9	13.5±4.7	C/R	-	-
	(69±30%)	(72±33%)	(65±20%)	(83±23%)	(78±26%)	(70±16%)	(72±29%)	(68±27%)	(58±12%)	H/R	-	-

Data presented as mean±SD. Normality was determined by Shapiro-Wilk (SW) test ($p>0.1$). Comparisons between baseline, end of bleeding [EndBld], and follow-up [FU] (mean over 7-hour follow-up) within groups were performed by paired t-test with Bonferroni's correction for multiple comparisons or Friedman's test with pairwise comparisons as *post-hoc* test as appropriate (according to SW). Comparisons between groups were performed by analysis of variance (ANOVA) with Fisher's least significance difference (FLSD) as *post-hoc* test or or Kruskal-Wallis (KW) with Conover-Iman as *post-hoc* test. PPT= partial thromboplastin time; PT= prothrombin time; INR= international normalized ratio; HGB=hemoglobin; HCT=hematocrit; RBC=red blood cells; WBC=white blood cells. Bld=(EndBld-Baseline), ΔFU=(FU-Baseline). $p<0.05$ considered significant and all tests 2-tailed. †$p<0.05$ vs baseline; ‡$p<0.05$ vs EndBld; [§]not significant by ANOVA/KW.

Supplemental Table S4: Sensitivity Analysis – Significant Results Without P108 and P109

Variable	Control (C)			HS (H)			RIPC+HS (R)			p value between groups		
	**Base**	**EndBld**	**FU**	**Base**	**EndBld**	**FU**	**Base**	**EndBld**	**FU**	**Comparison**	**ΔBld**	**ΔFU**
MAP [mmHg]	64±10	61±10	56±12	69±9	30±5†	38±11†	67±8	30±3†	42±9†‡	C/H	<0.001	0.002
										C/R	<0.001	<0.001
										H/R	NS	NS
HR [bpm]	89±12	82±12	93±18‡	83±9	107±27	127±31†‡	80±17	123±41†	164±26†‡	C/H	0.018	0.007
										C/R	0.002	<0.001
										H/R	**NS**	**0.008**
DO_2	458±154	462±142	459±150	557±72	235±80†	307±109†	500±108	246±67†	347±121†	C/H	<0.001	<0.001
										C/R	<0.001	0.018
										H/R	NS	0.087
ER	0.25±0.06	0.28±0.11	0.32±0.18	0.23±0.03	0.60±0.14†	0.53±0.15†	0.24±0.03	0.56±0.10†	0.42±0.10†‡	C/H	<0.001	0.047
										C/R	<0.001	0.004
										H/R	NS	NS

Data presented as mean±SD. Normality was determined by Shapiro-Wilk (SW) test ($p>0.1$). Comparisons between baseline, end of bleeding [EndBld], and follow-up [FU] (mean over 7-hour follow-up) within groups were performed by paired t-test with Bonferroni's correction for multiple comparisons or Friedman's test with pairwise comparisons as *post-hoc* test as appropriate (according to SW). Comparisons between groups were performed by analysis of variance (ANOVA) with Fisher's least significance difference (FLSD) as *post-hoc* test or Kruskal-Wallis (KW) with Conover-Iman as *post-hoc* test. MAP=mean arterial pressure, HR=heart rate, DO_2=Delivered Oxygen, ER=Oxygen Extraction Ratio; ΔBld=(EndBld-Baseline), ΔFU=(FU-EndBld), ΔFU=(FU-Baseline). $p\leq0.05$ considered significant and all tests 2-tailed. †$p\leq0.05$ vs baseline; ‡$p\leq0.05$ vs EndBld;

Summary of differences in sensitivity analysis:

1. MAP – no differences
2. HR – the difference between H/R during ΔBld - was significant before but not significant now
3. DO2 – change from EndBld to FU for R group - was significant before but not significant now
4. ER – difference between C/H at ΔFU- was not significant before (p=0.073) but significant now (p=0.047)
